# Mucous membrane pemphigoid with ocular involvement in a patient diagnosed previously with mucosal-dominant pemphigus vulgaris: A case report

**DOI:** 10.1016/j.jdcr.2025.06.041

**Published:** 2025-07-15

**Authors:** Celine Dan-Tam Nguyen, Jennifer Cao, Arturo R. Dominguez

**Affiliations:** a University of Texas Southwestern Medical School, University of Texas Southwestern Medical Center, Dallas, Texas; b Department of Ophthalmology, University of Texas Southwestern Medical Center, Dallas, Texas; c Departments of Dermatology and Internal Medicine, University of Texas Southwestern Medical Center, Dallas, Texas

**Keywords:** cicatrizing conjunctivitis, mucous membrane pemphigoid, mucus membrane pemphigoid, pemphigus vulgaris

## Introduction

This article reports the case of a patient with a history of biopsy-proven mucosal-dominant pemphigus vulgaris (PV) who later developed new ocular symptoms. A repeat biopsy confirmed the diagnosis of mucous membrane pemphigoid (MMP), and the patient was successfully treated with mycophenolate mofetil (MMF). This report discusses the clinical course, diagnostic challenges, and therapeutic interventions of this case.

## Case report

A 60-year-old woman with a medical history of Hashimoto’s thyroiditis, osteoporosis, and hypertension was diagnosed with PV after presenting with oral erosions and throat pain in 2010. She had no history of cancer, chemotherapy, or Stevens-Johnson syndrome. A biopsy from the right buccal mucosa revealed prominent suprabasal acantholysis, consistent with PV. Direct immunofluorescence (DIF) of the right lower lip demonstrated 1-2+ intercellular staining with IgG and C3 in the lower portions of the mucosa, characteristic of PV. There was no staining of C3, IgG, or IgA along the mucosal basement membrane zone (BMZ). Anti-desmoglein (DSG) 3 IgG enzyme-linked immunosorbent assay (ELISA) was elevated at 125.9 U/mL. Anti-DSG1 was negative. Initial treatment included oral corticosteroids and MMF up to 2000 mg/d. In 2011, she was transitioned to MMF monotherapy, but after failing a trial wean, her previous dosage was resumed.

**Fig 1:**
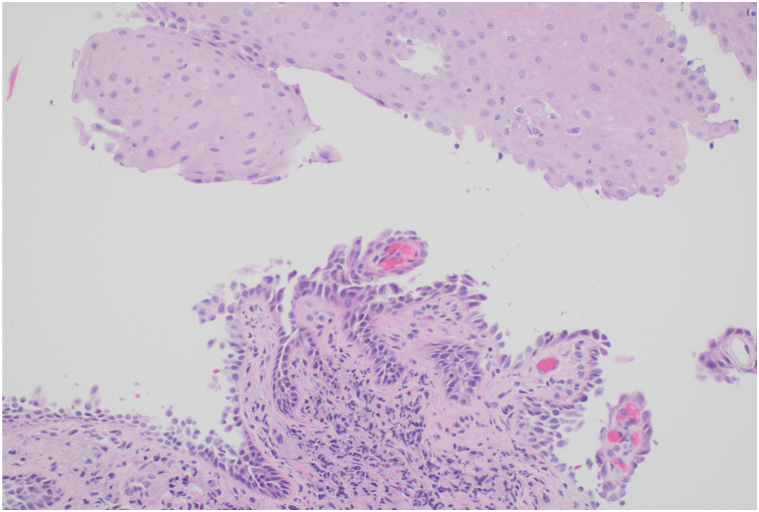
Epiglottal biopsy from September 2016 demonstrating suprabasal acantholysis (hematoxylin and eosin stain; original magnification: ×200.)

She first established care at our institution in July 2013, where she was stable on MMF 2000 mg/d. In February 2014, an attempt was made to taper the MMF to the lowest effective dose due to mild activity. Unfortunately, she flared with new lesions on her buccal mucosa, tongue, and gingiva, and anti-DSG3 IgG ELISA returned elevated at 43 U/mL. She was treated with rituximab 1000 mg × 2 in November 2014 and 500 mg in May 2015, leading to complete remission off all therapy. In July 2016, she developed globus sensation, pain, and cough, and histopathology of a biopsy from her epiglottis and right piriform sinus by otolaryngology revealed acantholysis consistent with PV (Fig 1). Serum anti-DSG3 IgG ELISA at that time was equivocal at 12 U/mL. Indirect immunofluorescence (IIF) microscopy of the monkey esophagus was negative for IgG. Because of concern that rituximab had provoked a non-ST elevation myocardial infarction following her last infusion, she was restarted on MMF 3000 mg/d instead. On this regimen, she was in partial remission with minimal oral disease and no ear, nose, and throat symptoms until May 2019, when she developed recurrence of throat pain and globus sensation. Repeat serum anti-DSG3 IgG ELISA was negative at 3 U/mL. After receiving clearance by her cardiologist, rituximab 1000 mg × 2 was repeated, and she received an additional 1000 mg 6 months later, leading to complete remission off all therapy.

**Fig 2:**
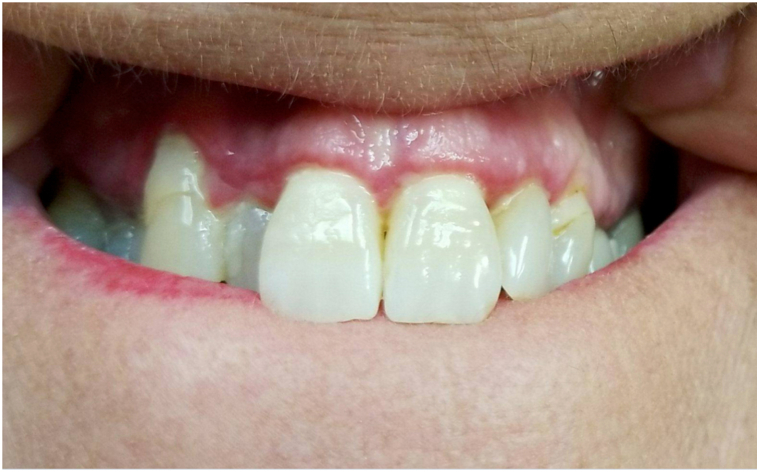
Erythematous superior gingival margin on March 2022 showing erythema along the gingival margin as well as gingival recession.

**Fig 3:**
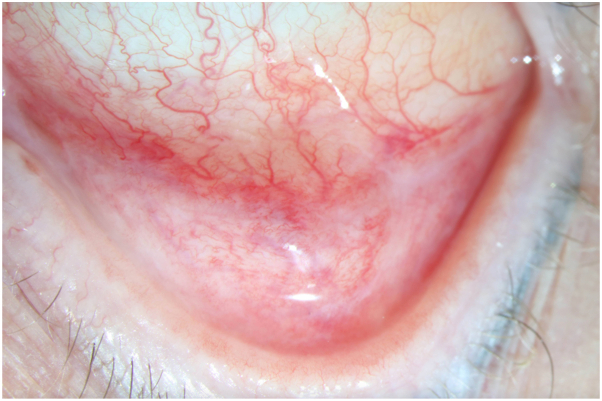
Left lower eyelid of patient in September 2023 demonstrates 2+ inflammation, subconjunctival fibrosis, and fornix foreshortening.

**Fig 4:**
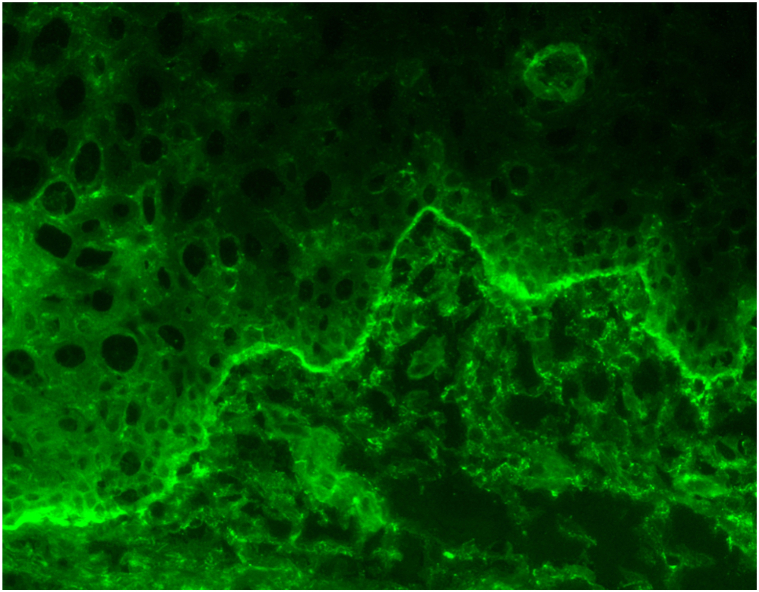
Buccal mucosal biopsy performed in September 2023. Direct immunofluorescent staining using fluorescein-rabbit F(ab’)2 anti-human IgG demonstrated 1+ continuous IgG staining at the basement membrane zone, suggestive of mucous membrane pemphigoid.

Three years later, she developed eye redness and dryness, initially diagnosed as allergic conjunctivitis. She had no history of ocular trauma. Despite treatment with serum tears, chondroitin eye drops, and montelukast 10 mg daily, her conjunctival redness worsened. She also noted to have persistent erosions and sensitivity of her gingiva (Fig 2). In August 2023, she was referred to an ophthalmologist (J.H.C.) to evaluate for pemphigus ocular involvement. Examination revealed 2+ conjunctival inflammation, subconjunctival fibrosis, and fornix foreshortening (Fig 3), findings more suspicious of MMP. A buccal mucosal biopsy was then performed for DIF, which demonstrated 1+ continuous IgG staining at the BMZ (Fig 4). There was no intercellular positivity of IgG or C3. Repeat serological testing included negative anti-DSG3, anti-BP-230, and anti-180 IgG ELISAs, as well as negative IIF microscopy studies of monkey esophagus and 1M NaCl split skin. We subsequently performed IIF testing on stored serum samples from 2015 and 2016, and both were negative for IgG and IgA on monkey esophagus and 1M NaCl split skin.

Based on her ocular findings and DIF, she was diagnosed with MMP with ocular involvement, and MMF 1000 mg was restarted. Her oral lesions completely healed by November 2023, and ocular inflammation was nearly resolved by January 2024. Because of gastrointestinal intolerance, MMF was transitioned to mycophenolic acid 1080 mg twice daily. She remains in remission at the time of publication.

## Discussion

PV and MMP are distinct autoimmune blistering disorders characterized by antibody targeting of adhesion molecules in the skin and mucous membranes.^1^ Ocular involvement in PV is uncommon, occurring in approximately 16.5% of patients and typically presents as conjunctivitis or corneal involvement.^2^ However, the development of cicatrizing conjunctivitis and scarring, particularly in long-standing disease, should prompt consideration of MMP, given its association with severe ocular morbidity.^3^ In our case, the patient initially presented with oral lesions typical of PV involving primarily her buccal mucosa and tongue, and later epiglottis, where biopsies for histopathology and DIF demonstrated features diagnostic of PV; the absence of BMZ involvement excluded MMP at that time. The development of persistent gingival erosions and new cicatrizing conjunctivitis necessitated reevaluation.^4^ Subsequent buccal biopsies revealed linear deposition of IgG and C3 at the BMZ on DIF, confirming MMP. Although conjunctival biopsies remain the gold standard for diagnosing ocular MMP,^5^ buccal biopsies can aid in diagnosing ocular cicatricial pemphigoid, particularly in cases with significant oral involvement.^6^

A 2001 review described 30 cases of dual PV and pemphigoid diagnoses, including 10 patients with both MMP and PV.^7^ However, at least 1 of these 10 patients had been included in a subsequently retracted study.^8^ Several other patients in this case series were authored by the same investigator implicated in the retraction, raising additional concerns about data validity.^9^ Other case reports, such as that by Kobayashi et al,^10^ documented simultaneous PV and MMP based on ELISA and immunoblotting but lacked confirmatory DIF or conjunctival biopsy. Kumar et al^11^ and Cram et al^12^ described patients with cicatricial pemphigoid who exhibited circulating intercellular antibodies, but without histologic evidence of PV, likely representing pemphigoid with pemphigus-like serologies rather than true dual pathology. Although some of these reports may reflect genuine overlap, many predate standardized diagnostic assays such as ELISA, immunoblotting, and split skin IIF and should be interpreted cautiously.

In contrast, our case uniquely demonstrates a biopsy-proven, temporally distinct clinical transition from PV to ocular MMP over a 13-year interval (Table I). From 2010 to 2015, our patient had multisite mucosal PV, affecting the buccal mucosa and tongue, with elevated anti-DSG3 serologies. In 2016, she developed epiglottal involvement where biopsy for histopathology demonstrated features consistent with PV (Fig 1). Beginning in 2018, her disease phenotype shifted, serologies remained negative, and clinical activity became limited to 1 to 2 gingival erosions per visit, without recurrence of extragingival or globus sensation. She was treated strictly with topical steroids. This gingival-limited pattern remained consistent through 2020 (Fig 2), and it is possible that at this point of her disease, the desquamative gingivitis represented MMP rather than PV, as patients with oral MMP are more likely to present with solely desquamative gingivitis, whereas those with PV are more likely to have nongingival involvement.^13^ It was during this time that she also developed ocular symptoms prompting reevaluation, and she was ultimately referred to ophthalmology. This sequential disease progression, rather than concurrent presentation, suggests a temporally mediated evolution, possibly mediated by chronic inflammation and epitope spreading.

**Table I:** Longitudinal timeline of clinical, serologic, and therapeutic findings

Date	Anatomic site(s) involved	Clinical description	DSG3 IgG ELISA (U/mL)	Indirect immunofluoresence	Treatment
2010 to July 2013	Buccal mucosa, tongue, and lips	Oral erosions	125.9 (Positive)		MMF 2000 mg/d
November 2014	Buccal mucosa, tongue, and epiglottis	Oral erosions	Not tested		Rituximab 1000 mg × 2 initiated
May 2015	Buccal mucosa and tongue	Oral erosions	43 (Positive)	Negative for IgG and IgA on monkey esophagus and 1M NaCl split skin.	Rituximab 500 mg MMF 2000 mg/d
March 2016	Buccal mucosa and tongue	Oral erosions	9 (Indeterminant)		MMF 2000 mg/d
September 2016∗	Epiglottis and left buccal mucosa	Biopsy confirmed PV of epiglottis	12 (Indeterminant)	Negative for IgG and IgA on monkey esophagus and 1M NaCl split skin.	MMF 3000 mg/d
January 2018	Gingiva and palatine tonsil	Oral erosions: gingiva-limited pattern begins	Negative (<20)		MMF 3000 mg/d
July 2018	Gingiva	1-2 gingival erosions; recession, bone loss	Not tested		MMF 3000 mg/d
December 2018	None	No oral erosions documented	Not tested		MMF 3000 mg/d
May 2019	Gingiva	1-2 gingival erosions; mild globus sensation	3 (Negative)		Rituximab 1000 mg × 2 initiated
September 2019	Gingiva	1-2 gingival erosions	<20 (Negative)		Rituximab 1000 mg maintenance
March 2020	Gingiva	1-2 gingival erosions	Not tested		Off systemic therapy (in remission)
August 2023†	Gingiva and conjunctiva	Gingival lesions; new ocular findings; buccal biopsy confirmed MMP	<20 (Negative)		MMF 2500 mg restarted
November 2023	None	Resolution of oral lesions	Not tested		MMF 1500 mg twice a day
January 2024	Conjunctiva	Near-complete resolution of ocular inflammation	Not tested		Switched to mycophenolic acid 1080 mg twice a day

Anti-DSG1, BP180, BP230 all negative at time of MMP diagnosis (August 2023). "Negative" DSG3 defined as <9 U/mL per laboratory reference.

*BMZ*, Basement membrane zone; *BP*, bullous pemphigoid; *Dsg*, desmoglein; *ELISA*, enzyme-linked immunosorbent assay; *MMF*, mycophenolate mofeti; *MMP*, mucous membrane pemphigoid; *NaCl*, Sodium chloride.

∗ Epiglottis biopsy confirmed PV with suprabasal acantholysis (Fig 1); DIF negative for BMZ involvement.

† Buccal biopsy confirmed MMP with linear IgG BMZ deposition (Fig 3); conjunctival inflammation evident (Fig 2).

Chronic inflammation in PV can expose previously hidden epitopes at the BMZ, thereby initiating an autoimmune cascade that targets newly exposed antigens.^14^^,^^15^ This phenomenon, termed epitope spreading, may play a pivotal role in the autoimmune transition from PV to MMP. Molecular evidence suggests that persistent mucosal inflammation in PV facilitates the progression from a primarily mucosal to a mucocutaneous phenotype, as observed with the formation of anti-DSG3 and anti-DSG1 antibodies.^14^^,^^15^ This autoantibody diversification contributes to the development of cutaneous blistering and extensive mucosal lesions.^14^^,^^15^ Notably, similar mechanisms of epitope spreading have been implicated in other autoimmune blistering disorders, such as ocular cicatricial pemphigoid secondary to Stevens-Johnson syndrome, in which antigenic release from chronic inflammation triggers a sustained autoimmune response against BMZ components.^16^^,^^17^ We also cannot discount the possible role of rituximab therapy in the transition from PV to ocular MMP. Previous literature has suggested that rituximab can be associated with a shift from a primarily Th1-dominant toward a more mixed Th1 and Th2 response, consistent with the inflammatory profile for pemphigoid as opposed to pemphigus. However, if this were the causative mechanism, one would expect more cases of PV to ocMMP transitions, given that rituximab is considered a gold standard treatment frequently used in modern PV therapy.

## Conclusion

The development of ocular symptoms in a patient with PV necessitated reevaluation and subsequent diagnosis of MMP. The presence of scarring, keratinization, and other ocular sequelae necessitates prompt intervention.^18^ Multidisciplinary collaboration among dermatologists, ophthalmologists, and pathologists is critical for the management of autoimmune blistering disorders.^4^ Future research into the mechanisms driving the transition between these conditions, enhancing diagnostic protocols, and improving targeted therapies will be central for optimizing patient outcomes in dermatologic practice.

## Conflicts of interest

None disclosed.
